# Continuous glucose monitoring in older people with diabetes receiving home care—a feasibility study

**DOI:** 10.1186/s40814-020-00754-3

**Published:** 2021-01-06

**Authors:** Annette Bævre Larsen, Monica Hermann, Marit Graue

**Affiliations:** 1 Department of Medicine, Akershus University Hospital, Kongsvinger, Norway; 2 Faculty of Health and Social Sciences, Institute of Health and Caring Sciences, Western Norway University of Applied Sciences, P.O. Box 7030, N-5020 Bergen, Norway

**Keywords:** Diabetes, Hypoglycemia, Older people, HbA1c, Home care

## Abstract

**Background:**

Hypoglycemic incidents in older people can cause severe health problems, enhance general age-related disabilities, and cause frailty. Little is known about incidences of hypoglycemia in older home-dwelling people with diabetes. Thus, the aim of this study was to examine the feasibility of capturing hypoglycemia and issues associated with increased risk of hypoglycemia by use of continuous glucose monitoring (CGM) and standardized questionnaires among older home-dwelling individuals with diabetes type 2 receiving home care.

**Methods:**

CGM with the Ipro2-blinded monitoring system were performed for 5 days in six home-dwelling individuals ≥ 75 years diagnosed with diabetes and receiving home care. Demographic (age, gender, living arrangements) and clinical data (diabetes diagnoses and duration, diabetes medication, documented treatment goal, available glycosylated hemoglobin (HbA1c)) were collected from electronic patient records in home care services. Feasibility (ease of use, quality of data, time spent) of standardized questionnaires to identify the risk of hypoglycemia (the McKellar Risk Assessment Tool), risk of malnutrition (the Mini Nutritional Assessment (MNA)), functional status (the Individual-based Statistics for Nursing and Care Services (IPLOS)), and cognitive status (the Mini Mental Status Exam (MMSE)) was also assessed. Questionnaire data was collected by a study nurse in the individuals’ home.

**Results:**

The practical use of CGM was satisfactory, with no major remarks about discomfort or technical errors, except for one participant with skin reaction (redness). Collecting data with the McKellar Risk Assessment Tool, MNA and IPLOS worked well according to quality of data, time spent, and ease of use. The MMSE survey required extensive training of personnel to be conducted.

**Conclusion:**

The feasibility study informs an upcoming study on the incidence and risk factors of hypoglycemia in home-dwelling older individuals. We will ascertain that personnel who will use the MMSE questionnaire to collect cognitive status and skills are familiar with the tool and adequately educated and trained before study start. The use of blinded CGM in this population was well tolerated and can be used “as is” for future studies.

## Key messages regarding feasibility

1. What uncertainties existed regarding the feasibility?
  Practical use of continuous glucose monitoring (CGM) among older home-dwelling individuals
  Feasibility of standardized questionnaires and ability of older people to complete
  Data collection in the individuals’ home
2. What are the key feasibility findings?
  Practical use of blinded CGM worked well
  Time spent and effort of personnel collecting data was reasonable
  Processing of data was viable with minor adjustment only
  Recruitment and trial procedures were acceptable
3. What are the implications of feasibility findings for the design of the main study?
  CGM can be used to detect occurrence of hypoglycemia in home care
  Personnel needs adequate education and training to use the MMSE instrument
  Software must be updated before study start of the main study

## Background

As a consequence of increased wealth and better treatment people with diabetes live longer [1]. In individuals receiving home care, a recent study conducted in Norway revealed a prevalence of 24% with diabetes [2, 3]. Glucose-lowering drugs are vital to maintain adequate blood glucose control and to prevent diabetes related comorbidity and complications. Several studies have shown that a substantial proportion of older people are treated too intensively with an HbA1c lower than recommended in guidelines for older people with diabetes, posing them at increased risk of hypoglycemia [4–6].

Hypoglycemic incidents in older people can cause severe health problems, enhance general age-related disabilities and cause frailty [7]. It is associated with a number of negative outcomes like vascular complications, cognitive dysfunction, risk of fall and fractures, and coma and death [8, 9]. In addition, response to hypoglycemia is impaired due to altered adaptive physiological response to low glucose levels in older people. Little is known about the prevalence of hypoglycemia among older people with diabetes and older home-dwelling people with diabetes, which represents a challenge for home care services in terms of patient safety.

WHO has defined patient safety as “prevention of errors and adverse effects to patients associated with health care” [10]. According to a recent literature review, there seems to be a gap between knowledge and understanding of patient safety and the risk of adverse events [11]. For people with diabetes living at home receiving secure health care, it is required that the health care personnel are able to identify symptoms to be able to take action.

Studies reporting on hypoglycemic incidents in older people with diabetes are often based on severe hypoglycemia in hospitalized patients, which does not describe the situation in a home-dwelling older population with diabetes [12–15]. Many factors that contribute to an increased risk of hypoglycemia, such as frailty, impaired cognitive function, poor nutritional status, and polypharmacy, increase with increasing age. In line with this, we have designed an observational study to investigate the feasibility of continuous glucose monitoring (CGM) and use of standardized questionnaires on issues associated with increased risk of hypoglycemia among older home-dwelling individuals ≥ 75 years with diabetes type 2 receiving home care [16]. The overall aim of the study was to examine the feasibility of capturing hypoglycemia and issues associated with increased risk of hypoglycemia by use of CGM and standardized questionnaires among older home-dwelling individuals ≥ 75 years with diabetes type 2 receiving home care. The findings of the present feasibility study will inform a larger observational study scheduled to autumn 2020.

### Research questions

We proposed the following research questions:

1. To what extent is the CGM Ipro2 system suitable for detecting occurrence of hypoglycemia in home-dwelling individuals ≥ 75 years with diabetes type 2 receiving home care?
2. To what extent are standardized questionnaires suitable for evaluating issues associated with increased risk of hypoglycemia, risk of malnutrition, level of functioning, and cognitive status in this study population?
3. To what extent are study procedures on collecting demographic, clinical, CGM, and questionnaire data suitable to study hypoglycemia in older people receiving home care with respect to ease for the individuals and health care professionals, quality of the data, and appropriateness of the data format for further processing?

## Methods

### Design

We used a feasibility study design to examine study procedures, as well as measures and instruments to be used in an upcoming larger observational study. The study does not focus on the outcome of the data itself [14].

### Setting and participants

We conducted the study in a municipality with approximately 5000 inhabitants in Eastern Norway during 7 weeks in November and December in 2018. The number of home-dwelling individuals with diabetes treated with glucose-lowering drugs in the municipality was approximately 230 individuals [17]. We identified all older home-dwelling individuals ≥ 75 years diagnosed with diabetes type 2 and receiving home care (*n* = 20). People who were unable to complete questionnaire data were excluded. Furthermore, we did not invite patients with severe cognitive deficiency (e.g., Alzheimer disease), severe medical comorbidity (e.g., end stage renal disease, severe heart failure, severe cancer), and/or a major psychiatric diagnosis (e.g., severe depression or bipolar disorder, schizophrenia) recorded in their medical records because the aim was to investigate adequate reactions to CGM measuring and questionnaire tools. We identified eligible (*n* = 9) participants from the home care electronic patient records asking them to participate. Three persons resigned due to severe illness or other circumstances. All participants signed a written consent form (*n* = 6). This sample size represents 10% of the calculated sample size in the main study and was therefore considered sufficient for the feasibility study.

### Data collection

Sociodemographic and clinical data from electronic patient records on date of birth, sex, height/weight, living arrangement (living with others, living alone), diabetes duration, diabetes medication (insulin and/or sulfonylurea), documented treatment goal (glycosylated hemoglobin (HbA1c)) (yes/no), frequency of HbA1c measuring, and type and incidence of adverse events. We developed a case report form for collecting this data.

### Blood glucose data

The two study nurses assisted by the first author (ABL) collected blood glucose data by connecting the CGM in the patients’ home. We used the Ipro2 CGM system (Medtronic MiniMed, Northridge, CA, USA) [18], a blinded glucose monitor. The Ipro2 monitor is a “silent” measuring tool which requires capillary blood glucose samples for calibration. For each participant, CGM data were collected for 5 consecutive days. In addition, capillary blood tests were performed three times daily during the 5 days either by the patient themselves or with help from the home care nurse when necessary. These capillary blood tests were used for calibration. The study nurses provided training for those participants who were able to fill in the meal diary and blood glucose measurement forms themselves.

### Questionnaire data

Complex disease, cognitive impairment, persistently low HbA1c, insulin/sulfonylurea treatment, hypoglycemia unawareness, and irregular or low food intake can increase the risk of hypoglycemic events. Therefore, we gathered data in order to capture the risk factors and compile them to the actual hypoglycemic events from CGM measurements. Practical issues in filling in questionnaires during home care visits, participants’ ability to complete the instruments, and time and need of resources was recorded. We also wrote down individual feedback from the participants who commented on the procedures, as well as the included items and scales within the instruments. We used the following standardized instruments to collect questionnaire data on (1) risk of hypoglycemia (high risk, score > 1p) (the McKellar Risk Assessment Tool), (2) risk of malnutrition (score > 17p) (the Mini Nutritional Assessment (MNA) scale), (3) level of functioning (the Individual-based Statistics for Nursing and Care Services (IPLOS), score > 3 need of help), and (4) cognitive status (the Mini Mental State Exam (MMSE), score < 25 cognitive impairment). Questionnaire data were collected on day 1 and 5 combining the connection and disconnection of the CGM sensor to save time.

*The McKellar Risk Assessment Tool* [19] represents questions about issues associated with increased risk factors on hypoglycemia, recognizing symptoms, mental state, treatment goals (in general HbA1c < 53 mmol/mol; however, for elderly frail people 64-70 mmol/mol), treatment (insulin/sulfonylurea), kidney or liver disease, recent episode, other medication, and nutrition [19]. *The Mini Nutritional Assessment scale* (MNA) [20] assesses the risk of malnutrition and is divided into two sections of questions, one short section (A–F) with a total score of 14 points where < 11 points constitutes “risk of malnutrition” and < 7 points is “malnutrition” [20]. Section two (G–R) gives an in-depth assessment to point out improvement areas with a total score of 16 points, a total score of 30 points all together. *Individual-based Statistics for Nursing and Care Services* (IPLOS) [21] focus on the individuals’ levels of functioning in interaction with their surroundings and are used in home care on a regular basis in Norwegian municipalities to verify need of assistance [21]. The variables are based on the International Classification and Functioning, Disability and Health (ICF) manual and represent different degrees of disability from 1 “no problems” to 5 “not able to”. *The Mini Mental State Exam* (MMSE) [22] is a diagnostic tool for cognitive status, impairment, and dementia [22]. It is divided into six eras of cognitive skills, each question derived 0 or 1 point.

### Analysis

We applied descriptive statistics for demographic and clinical data, number of invited participants, and number and percentage of people who agreed to participate. CGM data time-in-range (TIR) was defined between 3.9 and 8.3 mmol/L in this feasibility study. We calculated the percentage of total time in TIR for each individual. Furthermore, we calculated the total score for each individual as well as range of scores, for all questionnaire scales. For The McKellar Risk Assessment Tool [19] the total score is 12 where only one risk factor evaluates to “high risk of hypoglycemia” and two or more gives “very high risk of hypoglycemia”. The Mini Nutritional Assessment scale (MNA) [20] gives a maximum score of 30 points which indicates a “normal nutrition status”, < 24 points “risk of malnutrition”, and < 17 “malnutrition”. The Individual-based Statistics for Nursing and Care Services (IPLOS) [21] indicates a need for assistance if the score exceeds 3 points [21]. The Mini Mental State Exam (MMSE) [22] has a maximum score of 30 points. Normal functioning is 28–30 points, 25–27 points indicates cognitive impairment, and 24 points or less indicates cognitive failure, but further investigations are needed.

## Results

### Recruitment

**Fig. 1:**
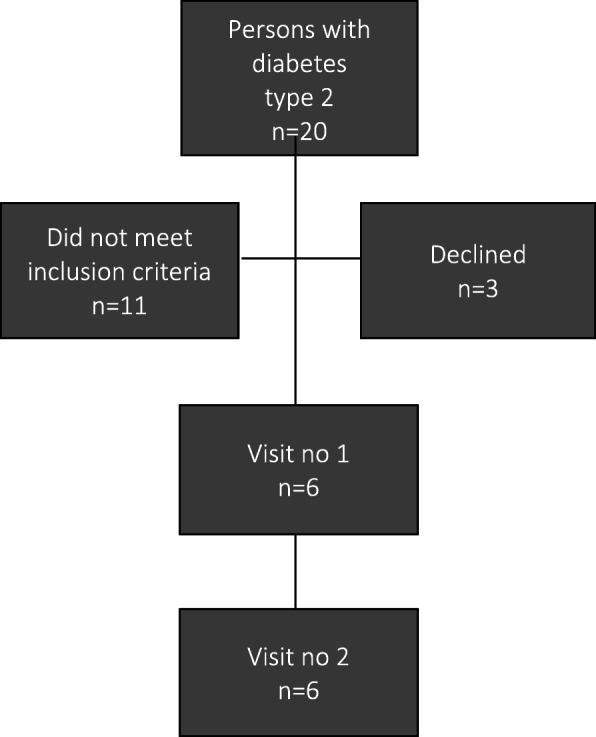
Flow chart—recruitment of study participants

In this small municipality, we identified 20 patients with diabetes type 2 of whom 12 were men and 8 women. Eleven of them did not meet the age inclusion criterion so we asked 9 eligible patients to participate. Two of them did not want to participate due to severe illness (cancer, mental illness) and one felt bound and stressed by the thought of having different people from home care entering their home several times a day. Finally, six participants agreed to participate (Fig. 1).

### Feasibility for collecting sociodemographic and clinical data

We collected data from EPJ in home care on sociodemographic and clinical data and treatment goals (HbA1c). Complete sociodemographic and clinical data were available for all patients. Lack of data was identified for HbA1c. The case report forms for collecting sociodemographic and clinical data, meal diary, and blood sugar measurements worked well.

### Sample characteristics

The participants had a duration of diabetes of 15 years or more. We included four males and two females and the median age was 82 (75–88) years. Three persons lived alone. All persons were treated with insulin and five individuals used additional glucose-lowering medication. Median BMI was 26.1 (23.9–30.7).

### Feasibility of CGM

**Fig. 2:**
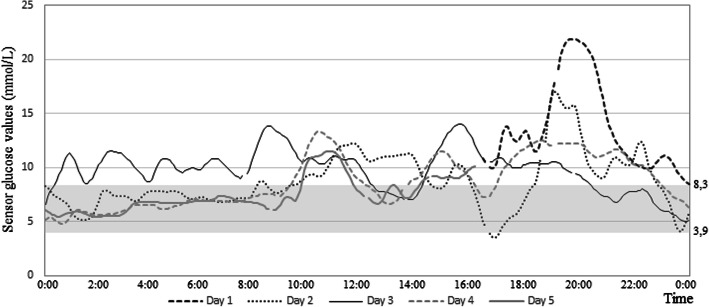
Example of 5 days continuous glucose monitoring (patient 5) (mmol/L)

The use of CGM among older home-dwelling people with diabetes was feasible. All patients completed all 5 days CGM measurement. Figure 2 displays an example of 5 days continuous glucose monitoring for one patient (patient 5).

Regarding practical use of CGM, one patient reported skin reaction (redness) and none reported pain. Furthermore, there were no technical errors, except for one sensor that fell off. Thus, the registration for this individual was delayed for 1 week. CGM data were obtained for a total of 29 days (1 day missing for one individual). Calibration samples were collected without any specific challenges. All individuals performed three measurements of capillary blood glucose daily, three of them had help from home care nurses who performed the tests. For 27 of the 29 days the mean absolute relative difference (MARD) was ≤ 15%, while for the remaining 2 days the MARD was < 20%. MARD is the average of the absolute error between paired samples, i.e., CGM values and corresponding capillary blood samples. Downloading the CareLink software to our computer was time-consuming due to compatibility problems with more recent drivers.

### Occurrence of hypoglycemia data

Regarding the CGM measurements, we collected 945–1140 sensor values between 3 and 22.2 mmol/L. TIR (3.9–8.3 mmol/L) varied between 0 and 63%. In three individuals, blood glucose values were < 3.9 mmol/L, i.e., hypoglycemic episodes, with a duration of between 15 and 50 min (Table 1).

**Table 1:** Continuous glucose measure values in older people (≥ 75 years) with diabetes receiving home care (*n* = 6)

	Person 1	Person 2	Person 3	Person 4	Person 5	Person 6
Sensor values (*n*)	1126	1091	1154	1138	1148	945
Highest (mmol/L)	19.4	12.5	22.2	18.4	21.8	22.2
Lowest (mmol/L)	6.8	3	5.7	8.3	3.5	3.1
Average (mmol/L ± SD)	11.7 ± 2.8	7.7 ± 1.8	14.1 ± 3.6	13 ± 1.7	9.2 + 2.9	14.1 ± 4.5
% within TIR^a^	10	63	7	0	45	9
% over 8.3 mmol/L	90	37	93	100	55	89
Under 3.9 mmol/L (*n*)	0	1	0	0	1	2
Duration under 3.9 mmol/L (min)	0	0:25	0	0	0:15	0:25/0:50

^a^Time-in-range

### Feasibility for questionnaires

**Fig. 3:**
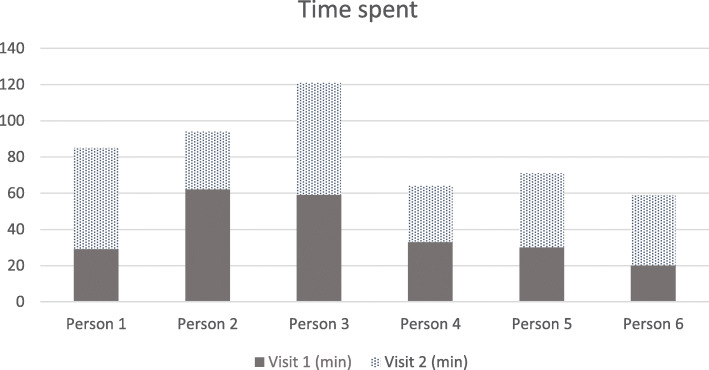
Data collection of older people (≥ 75) with diabetes type 2 receiving home care (*n* = 6)

Collecting questionnaire data during day 1 and 5 of the CGM period by two study nurses, assisted by the first author, was feasible. All patients completed the questionnaires. Some had difficulties understanding the meaning of some of the questions and we had to elaborate. As we assisted the patients in filling in the questionnaires, some people with hearing impairment had more difficulties which made the data collection more time-consuming. Median time spent on visit 1 was 32 (range 20–62) min versus 40 min on visit 2 (range 31–62) min (Fig. 3). In total, we spent less time on the last persons that were included due to better routines. Time spent with persons living with others or having relatives present during the interview was higher. Regarding practical use of the MNA scale and IPLOS, interview manuals were used to support the questionnaire (available online) [20, 21]. The protocol initially contained four questionnaires, but the MMSE required extensive skills and training among home care personnel and could therefore not be performed.

### Risk of hypoglycemia, malnutrition, and level of functioning

The McKellar Risk Assessment tool was suitable to identify factors associated with hypoglycemia. All six individuals were at “very high risk of hypoglycemia” due to risk factors like kidney disease, stroke, swallow palsy, diarrhea, insulin treatment (*n* = 5), and combination of insulin and sulfonylurea (*n* = 1) in addition to polypharmacy (*n* = 6). One person recently had a hypoglycemic event which required assistance. Regarding the McKellar risk survey, four out of six said that they did not notice any symptoms of hypoglycemia. Correspondingly, CGM measurements identified episodes of low blood glucose < 3.9 mmol/L in three of these four persons that did not notice any symptoms during the 5 days of data collection. In this questionnaire, one out of six reported HbA1c < 53 mmol/mmol and four out of six did not know their HbA1c level.

**Fig. 4:**
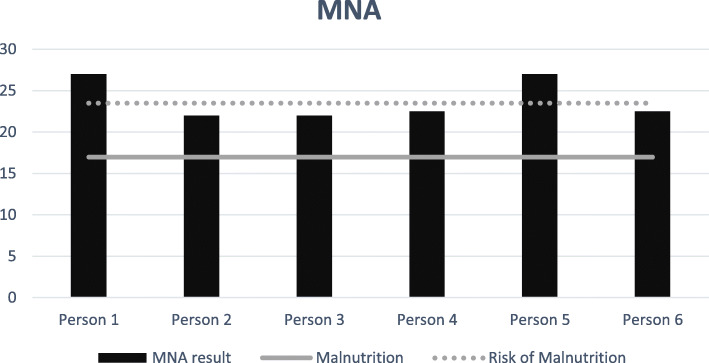
Risk of malnutrition in older people (≥ 75) with diabetes type 2 receiving home care (*n* = 6)

The Mini Nutrition Assessment was suitable to identify risk of malnutrition (Fig. 4). Four out of six persons had risk of malnutrition (score 17–23 points). Some persons had difficulty swallowing, others had a physical dysfunction caused by stroke, poor appetite from depression, esophageal hernia, and diarrhea and some forgot to eat because of their impaired cognitive functioning.

**Fig. 5:**
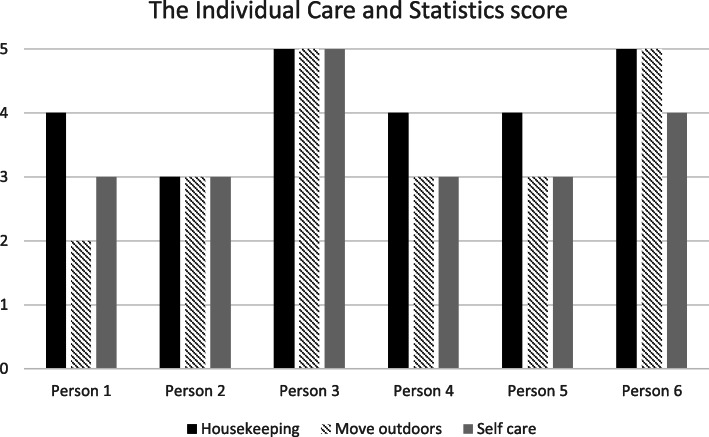
The Individual Care and Care Statistics in people (≥ 75) with diabetes receiving home care (*n* = 6). The Individual-based Statistics for Nursing and Care Services scores (IPLOS) (1 = no problems, 5 = not able to)

The Individual-based Statistics for Nursing and Care Services (IPLOS) was able to identify areas in physical dysfunctioning. The most common dysfunction areas were “common housekeeping”, “self-care”, and “moving outdoors” (Fig. 5). Common housekeeping gave the highest scores between 3 and 5 on (black) scale. Self-care (grey column) is the second highest score and several persons had disabilities (amputation of leg, leg wound, cognitive impairment/dementia) and were in need of medical attention and assistance. The third (shaded) column was moving outside which was difficult for all of them without the use of aids and assistance from others.

## Discussion

The use of CGM Ipro2 system to detect hypoglycemia and the use of standardized questionnaires collecting data to identify the risk of hypoglycemia, risk of malnutrition, and the individual’s level of functioning was feasible. Older home-dwelling people were willing to participate and found the study procedures safe and acceptable.

The results indicated that CGM was well suited to disclose the frequency and occurrence of asymptomatic hypoglycemia in older home-dwelling people with diabetes receiving home care. In research studies, the Ipro2 is used for blinded continuous glucose monitoring, as it does not have alarms and warnings in order not to interrupt the person’s regular blood glucose fluctuation to avoid hypoglycemia so that the person can make decisions to ingest carbohydrates before an incident occurs. Otherwise, in real monitoring, the warnings will serve as a counterbalance to avoid severe hypoglycemia and home care personnel will receive digital information and will be able to provide help. There are several studies which confirm that CGM is appropriate in monitoring the occurrence of asymptomatic hypoglycemia [23]. In previous research, it has been shown that hypoglycemic unawareness is highly prevalent in older adults and a common cause of silent hypoglycemia [8]. However, the use of CGM should be discussed in home care, as the technology identifies the occurrence of hypoglycemia that needs to be acted upon. This feasibility study has identified practical and technical aspects important for a larger observational study using CGM to identify hypoglycemia in home-dwelling older individuals receiving home care using insulin or other glucose-lowering drugs. The main study will inform on the actual frequency of hypoglycemia and thus provide important knowledge for the future use of CGM to prevent hypoglycemia in this population. Nevertheless, there is a question of cost, disturbing alarms, practical use, and training as well as organizational issues in home care service that need to be addressed.

Availability of study nurses during data collection was essential, as the participants needed some guidance to complete the questionnaires. Time spent in home visits was higher if the person had hearing problems and cognitive impairment. Moreover, using a clear voice, being well prepared, keeping eye contact, and acting calmly were important to build trust and minimize time spent. Collection of questionnaire data during home visits where the person lived together with a partner was also more time-consuming. Regardless, the partner provided good support and helped to create a safe atmosphere. To inform the main study, all questionnaires were feasible except for MMSE. The practical use of the McKellar tool, the MNA and IPLOS assessment worked well in the home care environment. However, using the MMSE was more challenging because of the requirement of trained personnel. This instrument demands more comprehensive training for study nurses to be able to perform. A recent review article highlights the gap between knowledge and understanding of safety for older people with diabetes in a home care setting [11]. The McKellar risk assessment tool identified three instances of hypoglycemia unawareness that can lead to serious complications and decreased safety, especially for those living alone.

Providing proper information about the study to all parties involved should be emphasized to optimize planning, procedures, and efficiency of measures. For two participants, a general practitioner had to be consulted to reconsider the dose of blood glucose-lowering medication. Therefore, to avoid misunderstandings and unnecessary time spent, formal collaboration with the participants’ general practitioners need to be established in the main study.

Optimal care requires correct and updated information about treatment goals (HbA1c) in addition to daily blood tests. Insufficient diabetes management routines with regard to regular assessment of HbA1c < 6 months in electronic patient records (*n* = 6) were identified. However, previous research has identified that a substantial proportion of older people treated with insulin or insulin secretagogues drugs are treated too intensively with an HbA1c lower than recommended, according to guidelines [2, 4–6]. Therefore, in the main observational study, this issue has to be addressed.

### Strengths and limitations

This study was conducted in only one small municipality with a limited number of participants (*n* = 6) which might differ from the variability among communities in the full scale study. Moreover, excluding cognitively impaired individuals may well exclude a very significant group of persons with diabetes in care who are at considerable risk of hypoglycemia. In addition, the study included only people using insulin and it gave no comparison to other diabetes treatments. Still, we included both genders and both persons who live alone and those with cohabitants. It might be considered a strength that the study nurses knew all participants which made the participants feel safe and well cared for. However, such close relations might also influence their answers to the questionnaires, e.g., they might appear more compliant regarding treatment and diet.

## Conclusion

The feasibility study informs an upcoming study on the incidence and risk factors of hypoglycemia in home-dwelling older individuals with diabetes. We will ascertain that personnel who will use the MMSE questionnaire to collect cognitive status and skills are familiar with the tool and adequately educated and trained before study start. The use of blinded CGM in this population was well tolerated and can be used “as is” for future studies.

## Data Availability

The datasets generated during the current study are available from the corresponding author on reasonable request.

## Abbreviations

CGM: Continuous glucose monitoring
EPJ: Electronic patient journal
MNA: Mini nutritional assessment
MMSE: Mini mental status exam
IPLOS: Individual-based statistics for nursing and care services
ABL: Annette bævre larsen
HbA1c: Glycated hemoglobin
TIR: Time-in-range
